# Bone Marrow-Derived Stem Cell (BMDSC) Transplantation Improves Fertility in a Murine Model of Asherman's Syndrome

**DOI:** 10.1371/journal.pone.0096662

**Published:** 2014-05-12

**Authors:** Feryal Alawadhi, Hongling Du, Hakan Cakmak, Hugh S. Taylor

**Affiliations:** 1 Department of Obstetrics, Gynecology and Reproductive Sciences, Yale University, New Haven, Connecticut, United States of America; 2 Department of Molecular, Cellular and Developmental Biology, Yale University, New Haven, Connecticut, United States of America; University of Udine, Italy

## Abstract

Asherman's Syndrome is characterized by intrauterine adhesions or fibrosis resulting as a consequence of damage to the basal layer of endometrium and is associated with infertility due to loss of normal endometrium. We have previously shown that bone marrow derived stem cells (BMDSCs) engraft the endometrium in mice and humans and Ischemia/reperfusion injury of uterus promoted BMDSCs migration to the endometrium; however, the role of BMDSCs in Asherman's syndrome has not been characterized. Here a murine model of Asherman's syndrome was created by traumatizing the uterus. We evaluate stem cell recruitment and pregnancy after BMDSCs transplantation in a model of Asherman's syndrome. In the Asheman's syndrome model, after BMDSC transplant, the Y chromosome bearing CD45-cells represented less than 0.1% of total endometrial cells. Twice the number of Y+CD45- cells was identified in the damaged uterus compared to the uninjured controls. There was no significant difference between the damaged and undamaged uterine horns in mice that received injury to a single horn. In the BMDSC transplant group, 9 of the 10 mice conceived, while only 3 of 10 in the non-transplanted group conceived (Chi-Square p = 0.0225); all mice in an uninjured control group conceived. The time to conception and mean litter size were not different between groups. Taken together, BMDSCs are recruited to endometrium in response to injury. Fertility improves after BMDSC transplant in Asherman's Syndrome mice, demonstrating a functional role for these cells in uterine repair. BMDSC transplantation is a potential novel treatment for Asherman's Syndrome and may also be useful to prevent Asherman's syndrome after uterine injury.

## Introduction

Asherman's Syndrome is characterized by intrauterine adhesions (synechiae) or fibrosis resulting as a consequence of damage to the basal layer of endometrium. This disease often leads to hypomenorrhoea or amenorrhoea, infertility or recurrent pregnancy loss. [1] Uterine synechiae have recently been associated with a significant increase in the risk of preterm premature rupture of membranes (PROM), placental abruption, and malpresentation. [2]

Asherman's syndrome occurs most commonly as a result of trauma or infection, particularly after pregnancy when estradiol levels are low. It occurs in up to 20–25% of patients treated with dilatation and curettage 1–8 week's post-partum. [3]–[4] The clinical symptoms depend on the degree and the localization of endometrial damage in the uterine cavity. The goal of Asherman's syndrome treatment is to re-establish a normal uterine cavity and restore uterine function. The current approach uses hysterocopic surgery to remove adhesions, and hormonal therapy to regenerate the endometrium from residual progenitor cells. [5] However, severe damage to the basal layer may not be amenable repair due to loss of most endometrial cells; the endometrium may fail to regenerate. In severe cases, treatment is difficult and the prognosis is usually poor.

The presence of endometrial epithelial and stromal colonies with high proliferative potential provides evidence for the existence of putative endometrial progenitor stem cells [6].The progenitor cells residing in the basalis layer of endometrium are thought to serve as a source of endometrial regeneration. [7]–[8] However, in the setting of severe damage to the basal layer of endometrium, the local endometrial progenitor stem cells may be destroyed and lose the ability to regenerate the endometrium.

Bone marrow-derived stem cells (BMDSCs) have been shown to travel to distant organs and contribute to tissue repair and regeneration [9]. BMDSCs have been detected in both human and mouse uterine endometrium, suggesting that BMDSCs are potential endometrial stem cells that may serve as a source of reparative cells for the reproductive tract [10]–[11]. Local injury signals likely play an important role in mobilization of BMDSCs to injured tissues. We have recently shown that Ischemia/reperfusion injury to the uterus promotes BMDSCs migration to and engraftment in the endometrium; BMDSCs are recruited to the endometrium in response to injury. [12] However, the role of BMDSCs in Asherman's syndrome has not been previously characterized. Here a reproducible murine model of Asherman's syndrome was created by traumatizing the uterus. We evaluated stem cell recruitment and the potential for functional improvement in Asherman's syndrome with the use of BMDSCs transplantation.

## Materials and Methods

### Animals

B57BL/6 male and female mice were obtained from Charles River Laboratories (Wilmington, MA) and maintained in the Animal Facility of Yale University School of Medicine. Mice are housed four to five per cage in a room with a 12 hr light/dark cycle (7:00 am–7:00 pm) with *ad libitum* access to food and water. All animals were treated under an approved Yale University institutional animal care and use committee protocol. Animal handlers were blinded to the experimental group. Tissues obtained were examined in a blinded fashion.

### Murine experimental asherman's syndrome

8-week-old C57BL/6female mice were used to create Asherman's syndrome mouse model. [13] Briefly, after administration of ketamine/xylazine by intraperitoneal injection, a vertical incision was made in the abdominal wall and the uterus exposed. A small incision was made in the each uterine horn at the utero-tubal junction and the horn traumatized in a standardized fashion using 27 Gauge needle inserted two-thirds of the way through the lumen, rotated and withdrawn four times. In initial experiments conducted to evaluate fibrosis without bone marrow transplant, a total of 12 mice were randomized to uterine damage or abdominal surgical incision alone. Three estrous cycles after uterus damage, the uterine horn were collected. The formalin-fixed paraffin-embedded uterine tissue was sectioned longitudinally and stained with hematoxylin and eosin (H&E) as well as Trichrome stain for histological evidence of fibrosis. Photomicrographs were taken using an Olympus BX41 microscope.

### Bone marrow cell isolation and transplantation

Donor BM was flushed from the femurs, tibias, and humeri of 8-week-old C57BL/6 male mice with cold sterile phosphate-buffered saline (PBS). The marrow suspension was filtered through sterile 70- µM Nitex mesh (Becton, Dickinson and Company). Twenty C57BL/6 female mice underwent induction of experimental Asherman's syndrome in both uterine horns and subsequently randomized into two groups. Immediately after the uterine damage, 1×10^7^ unfractionated BM cells in 200 ul saline were transplanted by tail vein injection in one group. The non-BM transplant group was injected with normal saline. Ten female mice were used as control and did not undergo uterine damage or BM transplant. After three estrous cycles the female mice were bred with C57BL/6 male mice for a period of 3 months. The number pregnant, time to conceive and litter size were recorded. Another ten mice underwent uterine damage, BM transplantation and tissue collection after 3 months; uterine sections were cut longitudinally, stained with H&E to evaluate the histological evidence of fibrosis and evaluated by Y chromosome FISH and CD45 immunofluorescence.

### Single uterine horn injury model

8–week-old C57BL/6 female mice were administered intraperitoneal injections of busulfan (Sigma) and cyclophosphamide monohydrate (Sigma) for 7 days in row, following an established protocol [14]. Approximatly1×107 unfractionated BM cells were transplanted by internal jugular vein injection after myeloablation. Two weeks after BM transplant, the mice were randomly divided into two groups. In the injury group, the damage was created as described above. The left horn of uterus was untouched. In the control group, identical abdominal surgical incisions were created; however, neither horn was damaged. Three months after uterine horn injury, uteri were collected and stained by Y -chromosome FISH, CD45 and cytokeratin immunofluorescence stain.

### Y FISH and immunofluorescence

As previously described [11]–[12], Y FISH stain was performed on Formalin-fixed paraffin-embedded specimens. Tissue were cut into serial 3- µm-thick sections, placed on coated slides, and deparaffinized through a series of xylene and ethanol washes. For Y FISH, CD45, and cytokeratin analysis, sections were incubated in Retrievagen A solution (BD Biosciences) for 30 min at 100°C and then 20 min at room temperature. Y FISH was performed using a digoxigenin-labeled Y chromosome probe and anti-digoxigenin-rhodamine antibody (Roche Diagnostics). The digoxigenin-labeled murine Y probe was kindly supplied by Dr. Diane Krause (Yale University). After Y FISH, slides were incubated simultaneously with both 1∶20 rat anti-mouse CD45 (BD Biosciences) and 1∶100 rat anti-mouse F4/80 (eBioscience, Inc.) at 4°C overnight followed by 1∶500 anti-rat-Alexa 488 (Molecular Probes) for 1 h at 37°C. Alternatively, slides were incubated with 1∶100 anti-cytokeratin (Z0622; Dako) or 1∶30 anti-vimentin (Cell Signaling) at 4°C overnight, followed by 1∶500 anti-rabbit-Alexa 647 (Molecular Probes) for 1 h at 37°C. All slides were coverslipped using Vectashield/4, 6-diamidino-2-phenylindole (Vector Laboratories). Negative and positive controls for Y chromosome consisted of sections of uterus from female-to-female BM transplants and testis, respectively. Negative and positive control tissues were processed simultaneously in each staining run. For each cell type, at least 100,000 cells were counted each uterine horn.

### Tissue analysis, cell counts, and image capture

BM-derived epithelial and stromal cells were counted by systematically examining the slides under ×40 magnification using an Olympus BX-51 microscope (Olympus). Areas of endometrium were randomly chosen and full-thickness counted. Multiple random slides of uterine sections from each mouse uterus were stained for use. Photomicrographs were taken using an Olympus BX51 fluorescence microscope. Images were captured with a digital camera, using IPLab software (BD Biosciences, www.bdbiosciences.com). Images were obtained using appropriate excitation and emission filter sets for 4′,6′-diamidino-2-phenylindole (nuclei), rhodamine (Y chromosome), fluorescein isothiocyanate (CD45, F4/80), and Cy5 (cytokeratin and vimiten). For quantitative trichrome analysis, five random sections of trichromatic slides were electronically scanned into an RGB image at magnification of 400X, which was subsequently analyzed using Image J (version 1.32j) software (National Institutes of Health, USA http://rsb.info.nih.gov/ij/). The amount of fibrosis (density and area) was then estimated from the RGB images. [15] Quantitative of trichromatic sections were assigned in a double-blinded way by two people. Data presented are mean ± standard error of the mean. The Mann-Whitney Rank Sum test was used to compare the data.

## Results

### Histological evidence of fibrosis in mouse model of Asherman's Syndrome

**Figure 1:**
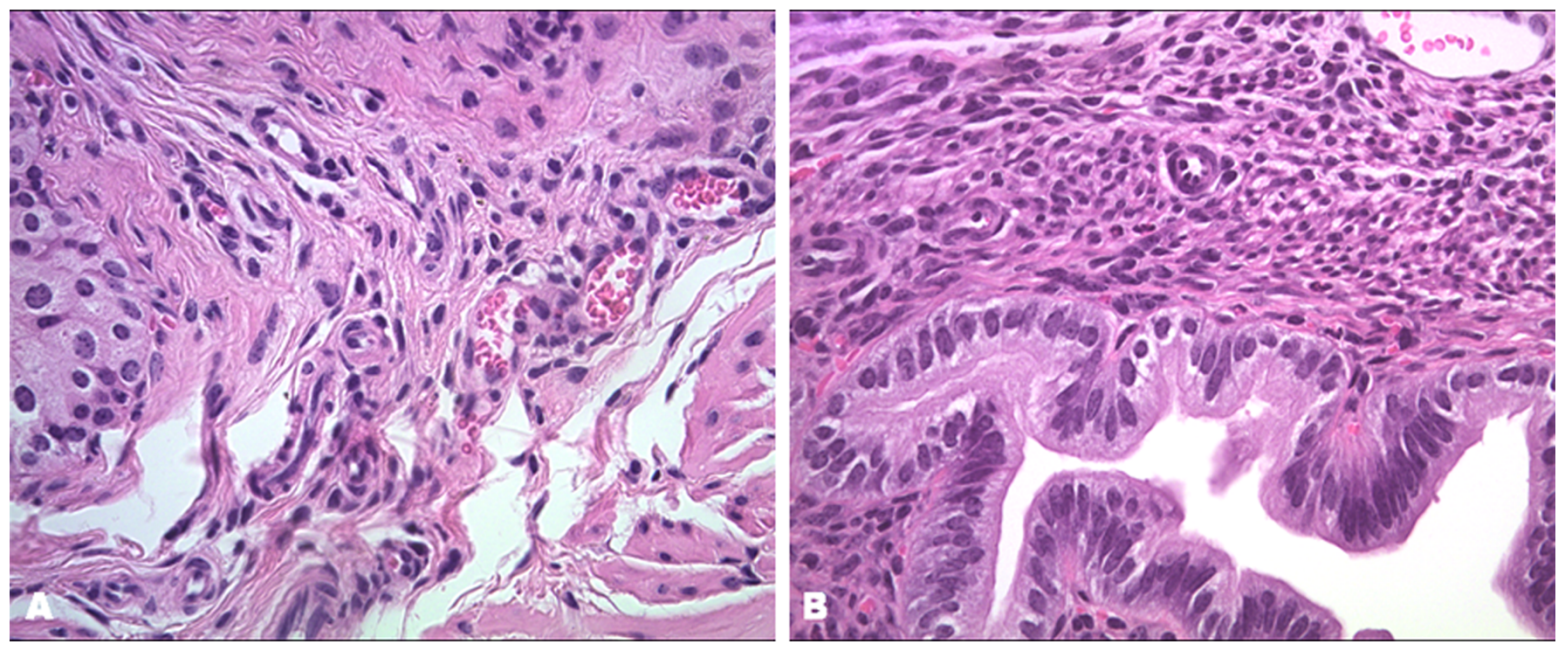
Histology of murine experimental Asherman's syndrome. Three estrous cycles after uterus damage, the uterine horns were collected and stained with hematoxylin and eosin (H&E). (A) Fibrosis was observed in the damaged uterei. (B) No fibrosis was seen in uteri from control group. Original magnification, 400X.

**Figure 2:**
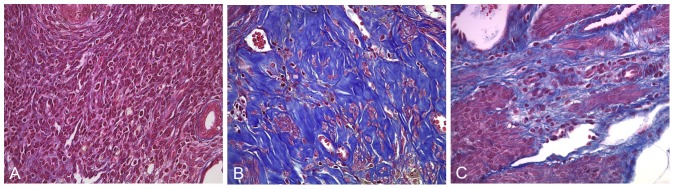
Trichrome stain of fibrosis of murine experiment Asherman's syndrome. The mice uterine horns were collected from the control group, experimental Asherman's syndrome group and BMT after uteri damage group. (A) No fibrosis was seen in uteri from the control group. (B) Fibrosis was observed in the Non- transplanted Asherman's group. (C) Fibrosis was also seen in BM-transplanted Asherman's group. Many animals in the treated group showed significantly decreased fibrosis. Original magnification, 400X.

To evaluate the murine Asherman's syndrome model, the uterine horns were collected three estrous cycles after uterine damage, and stained with hematoxylin and eosin (H&E). In all traumatized mice, the histologic evidence of fibrosis was confirmed in the uterus. (Figure 1) Fibrosis varied from 40% to 80% in each mouse. No significant difference in fibrosis was observed between the control and BM transplant group based on H&E staining. To evaluate the influence of BM transplantation on uterine damage, mouse uteri were collected 3 months after BM transplantation and stained with H&E and with trichrome for the histological evidence of fibrosis. Trichrome staining confirmed fibrosis in all traumatized mice. (Figure 2) There was no significant difference in fibrosis between BM-treated group (45.036±12.057) and the untransplanted group (49.405±10.244) (p = 0.535). The significant overlap in the level of fibrosis between the BM-treated and the untransplanted group, suggested that altered fibrosis alone was the enough account for the consistent changes in uterine function described below.

### BMDSCs engraft the uterus in the mouse model of Asherman's Syndrome

To evaluate BMDSCs recruitment in Asherman's syndrome, damaged uteri were collected three months after BM transplantation, and stained using Y chromosome FISH. Testis was used as a positive control and demonstrated approximately 85% of the cells staining positive for Y chromosome. CD45, a pan-white cell marker, was used here to distinguish endometrial cells from transient leukocytes present in the endometrium. F4/80 is a macrophage-specific marker simultaneously used to assure the ability to distinguish endometrial cells. As expected, no Y chromosome positive cells were observed in those mice that did not undergo transplant. In the mice with uterine damage and BM transplant, the average Y chromosome signal was 92.1 ±12.4/10,000 cells in each uterine horn. The Y chromosome bearing cells (BMDSCs) represented approximately 0.1% of the total number of endometrial cells.

### Localized uterine damage recruits BMDSCs throughout the endometrium

**Figure 3:**
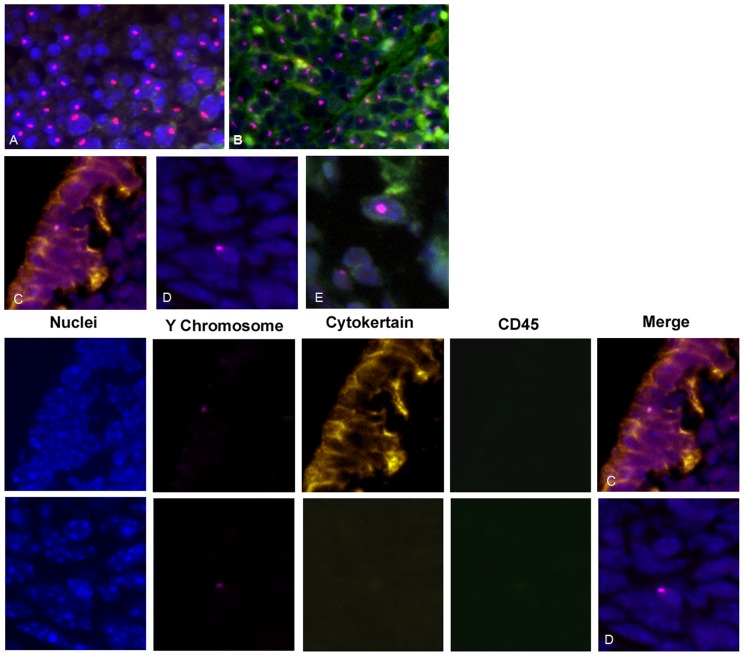
Localized uterine damage recruits BMDSCs throughout the endometrium. Immunofluorescence staining of Y chromosome (red) demonstrates male donor-derived BM cells localized in the endometrium of female recipients. Immunofluorescence staining of CD45 (green) distinguishes endometrial cells from leukocytes. Immunofluorescence staining of cytokeratin (yellow) indicates differentiated epithelial cells. DAPI is used to stain nuclei (blue). Top Panel: (**A**) Y chromosome signal in the testis shown as a positive control. (**B**) Y chromosome signal in male spleen tissue demonstrating expression of CD45 (pan leukocyte marker). (**C**) Y chromosome-positive, cytokeratin-positive, and CD45-negative cell, demonstrating a BM-derived endometrial epithelial cell.(D) Y chromosome-positive, cytokeratin-negative, and CD45-negative cell, demonstrating a BM-derived endometrial stromal cell in the endometrium (**E**) Y chromosome-positive, CD45-positive cell, demonstrating a transient leukocytes present in the endometrium. Lower Panel: Individual fluorescence images that result in merged figures 3 C and D from top panel. DAPI is used to stain nuclei (blue), Y chromosome FISH (red), immunofluorescence staining of cytokeratin (yellow) and CD45 (green) Original magnification, 400X.

To investigate the influence of the endometrial injury on migration of BMDSCs to a single uterine horn, in separate experiments one horn was damaged. Both the damaged and the undamaged horns were collected three months after BM transplantation. In formalin-fixed paraffin-embedded uterine tissue sections, the Y chromosome was visualized with the DIG-labeled murine Y probes. (Figure 3) We detected an average of 1/2600 Y+CD45- cells in control verses 1/1400 Y+CD45- cells in the damaged right horn and 1/1600 in the undamaged left horn (both P<0.001 compared to control) (*t test*, SigmaPlot 11.0). There was no significant difference between right and left uterine horns. The results by horn and cell type are summarized in table 1.

**Table 1:** Number of Y+CD45- stem cells recruited/10,000 total cells.

	Stromal cells N/10,000(±SEM)	Epithelium cells N/10,000(±SEM)	Total cells N/10,000(±SEM)
Control (n = 4)	27±2.082	13±0.577	40±2.646
Damage Right Horn (n = 3)	55±3.786*	14±1.528	71±3.512*
Undamaged (n = 3)	50±4.163*	14±2.646	63±2.517*

*P<0.001 control vs. each horn; there was no significant difference between the right and left horns.

### Pregnancy outcome

**Figure 4:**
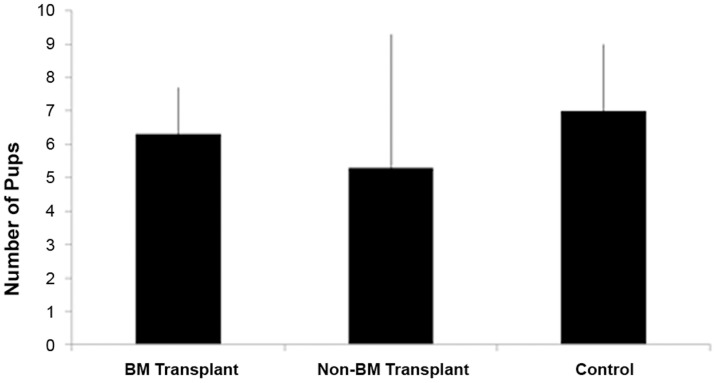
Average litter size. The average number of pups born is shown. (A) BM transplant Asherman'sgroup; (B) Non-BM transplant Asherman's group; (C) Control group. There is no significant difference between groups.

To investigate the functional improvement in Asherman's syndrome after BMDSCs transplantation, the female mice were bred starting in the third estrous cycles after BM transplantation for a period of three months. In the BM transplant group 9 of the 10 mice conceived, while only 3 of 10 in the non-BM transplant group conceived (Chi-Square p = 0.0225) (Chi-Square test, SigmaPlot 11.0). (Table 2) In the control group without uterine injury, 10 of the 10 mice conceived. The mean litter size was 6.3±1.4 in the BM transplant group, 5.3±4.0 in the non-BM transplant group and 7.0±2.0 in control group. (Figure 4) There were no significantly differences between groups (p>0.05). There was also no significant difference in the time to conception between groups.

**Table 2:** Conception Rate.

	Pregnant	Not Pregnant
BM Transplant	90%	10%
Non-BM Transplant	30%*	70%

*P  = 0.022 Non-BM transplant vs. BM transplant group.

## Discussion

Asherman's syndrome is associated with infertility due to loss of normal endometrium. The prevalence of Asherman's syndrome is not well defined, however, a retrospective case series reported that this disease is found in 13% of women undergoing routine infertility investigation.[16] Treatment of Asherman's syndrome is not consistently successful; therapies are limited to surgical restoration of the uterine cavity. The outcome of surgical therapy relies on the ability of the endometrium to regenerate after uterine patency is restored. Unfortunately endometrial growth in women with Asherman's syndrome is inconsistent. [17]–[18] The inability to regenerate a new endometrium likely reflects the loss of the endometrial basalis.

In Asherman's syndrome, severe damage to the basalis may destroy the local endogenous progenitor stem cells. Our previous studies have shown that BMDSCs are recruited to the endometrium and can engraft the endometrium. Recently we have demonstrated that stem cell recruitment is increased in response to injury. [12] Recruitment was independent of the existence of an estrus cycle or hormonal stimulation. Stem cell flux to the uterus is likely a reparative mechanism in response to injury or pregnancy rather than a means to replace endometrial cells lost by menstruation. [12] In fact, a recent study suggests that male bone marrow-derived stem cells may not contribute at all. [19] The number of BMDSCs that engraft the uterus is low, and these cells to not undergo clonal expansion to replace the entire endometrium; rather, they likely secrete trophic factors that aid in uterine repair and regeneration. [20]–[23] In the setting of overwhelming injury, as is seen in severe Asherman's syndrome, the constrained supply of stem cells may be the limiting factor in the repair process. A diminished stem cell pool or altered ability to recruit them to the uterus likely contributes to the risk of Asherman's syndrome.

To investigate the possibility that BMDSCs could regenerate the endometrium in Asherman's syndrome, we mimicked the pathophysiology of Asherman's syndrome in a mouse model. We created a reproducible model of Asherman's syndrome and demonstrated fibrosis in the damaged uterine horn. After uterine injury and male BM transplant, Y+ cells were detected in the BM treatment group. After BM transplant 9 of the 10 mice conceived, while only 3 of 10 in the non-BM transplant group conceived. These data suggest that treatment with BMDSCs improves fertility after uterine injury. The limited supply of reparative stem cells may a limiting factor in repair of the uterus after extensive injury. An enhanced supply of stem cells likely promotes endometrial regeneration and restores fertility.

Localized uterine horn damage recruits BMDSCs to the endometrial stroma of both uterine horns. Inflammation and injury may play an important role in the recruitment BMDSCs to endometrium. It is likely a secreted signal that can attract stem cells to the uterus rather than a local effect recruiting stem cells specifically to the site of injury within the uterus. Even a small area of damage may be useful to recruit stem cells to the uterus and could explain the reported success of endometrial biopsy in enhancing IVF success rates.

Here we demonstrate that BMDSCs play a functional role in the regeneration of endometrium. Deficient BMDSCs recruitment may contribute to Asherman's syndrome and modulation of this process offers a potential a novel therapy for this disease.

## Supporting Information

Figure S1 Immuniohistochemical analysis of cytokeratin expression. (A) Control group. (B) Non-BM transplant Asherman's group; (C) BM transplant Asherman's group. There is no significant difference between groups.
(TIF)
