# Supplementary figures and images for: Bone Marrow-Derived Stem Cell (BMDSC) Transplantation Improves Fertility in a Murine Model of Asherman's Syndrome
Source: PLoS One. 2014 May 12;9(5):e96662. doi: 10.1371/journal.pone.0096662 (PMC4018329; doi:10.1371/journal.pone.0096662)

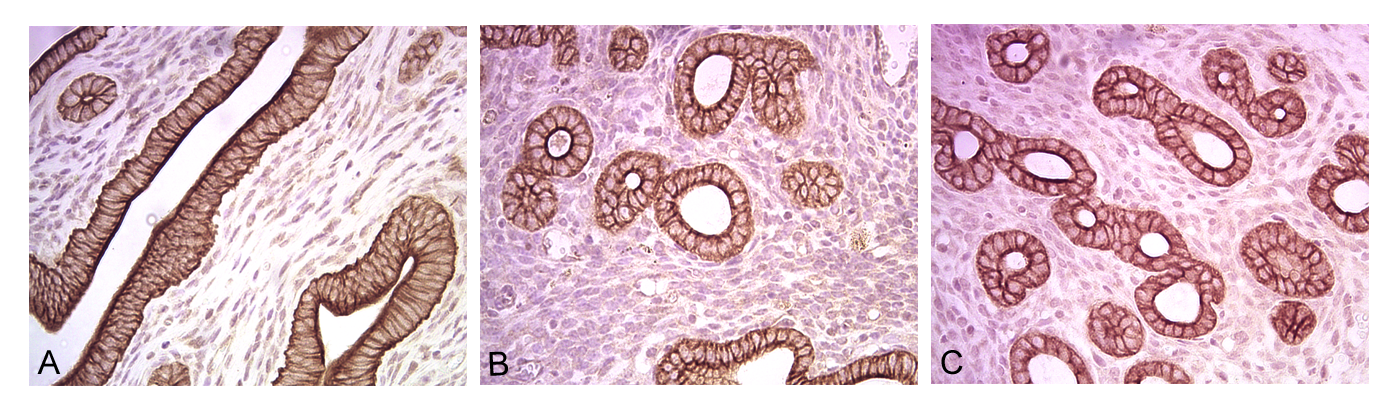

Supplement: Figure S1 — Immuniohistochemical analysis of cytokeratin expression. (A) Control group. (B) Non-BM transplant Asherman's group; (C) BM transplant Asherman's group. There is no significant difference between groups. (TIF) [file pone.0096662.s001.tif]
